# Association of CNVs with methylation variation

**DOI:** 10.1038/s41525-020-00145-w

**Published:** 2020-09-24

**Authors:** Xinghua Shi, Saranya Radhakrishnan, Jia Wen, Jin Yun Chen, Junjie Chen, Brianna Ashlyn Lam, Ryan E. Mills, Barbara E. Stranger, Charles Lee, Sunita R. Setlur

**Affiliations:** 1 Department of Bioinformatics and Genomics, College of Computing and Informatics, University of North Carolina, Charlotte, North Carolina 28223 USA; 2 Department of Pathology, Brigham and Women’s Hospital and Harvard Medical School, Boston, Massachusetts 02115 USA; 3 Department of Computational Medicine and Bioinformatics, University of Michigan, Ann Arbor, Michigan 48109 USA; 4 Department of Pharmacology, Northwestern University, Chicago, Illinois 60611 USA; 5 The Jackson Laboratory for Genomic Medicine, Farmington, Connecticut 06032 USA; 6 Department of Life Sciences, Ewha Womans University, Seoul, 03760 South Korea; 7 Precision Medicine Center, The First Affiliated Hospital of Xi’an Jiaotong University, Xi’an, 710061 Shaanxi China; 8 Present Address: Department of Computer and Information Sciences, College of Science and Technology, Temple University, Philadelphia, Pennsylvania 19122 USA

**Keywords:** Genetic variation, DNA methylation

## Abstract

Germline copy number variants (CNVs) and single-nucleotide polymorphisms (SNPs) form the basis of inter-individual genetic variation. Although the phenotypic effects of SNPs have been extensively investigated, the effects of CNVs is relatively less understood. To better characterize mechanisms by which CNVs affect cellular phenotype, we tested their association with variable CpG methylation in a genome-wide manner. Using paired CNV and methylation data from the 1000 genomes and HapMap projects, we identified genome-wide associations by methylation quantitative trait locus (mQTL) analysis. We found individual CNVs being associated with methylation of multiple CpGs and vice versa. CNV-associated methylation changes were correlated with gene expression. CNV-mQTLs were enriched for regulatory regions, transcription factor-binding sites (TFBSs), and were involved in long-range physical interactions with associated CpGs. Some CNV-mQTLs were associated with methylation of imprinted genes. Several CNV-mQTLs and/or associated genes were among those previously reported by genome-wide association studies (GWASs). We demonstrate that germline CNVs in the genome are associated with CpG methylation. Our findings suggest that structural variation together with methylation may affect cellular phenotype.

## Introduction

The extent of genetic variation that exists in the human population is continually being characterized in efforts to identify genetic factors that contribute to disease and evolution. Inter-individual genetic variation comprises primarily single-nucleotide polymorphisms (SNPs) and copy number variants (CNVs), the latter including gains and losses of DNA spanning >1 kb. The HapMap Project^1^ and the 1000 Genomes Project^2–4^ generated detailed maps of common genetic variants within and between human populations. However, the extent of the influence of CNVs^2,4–7^, the more recently appreciated class of large-scale germline variants, on gene function and phenotype remains under-characterized.

CNVs can regulate transcript expression either directly by overlapping gene-coding sequences or indirectly by altering regulatory non-coding regions. The role of non-coding regions in regulation of gene expression has been highlighted by the series of investigations from the ENCODE Consortium^8^. These studies have demonstrated that non-coding regions are replete with regulatory sequences such as transcription factor-binding sites (TFBSs) and enhancer sequences. Given these observations, it can be hypothesized that genetic variants in non-coding regions can potentially affect the functionality of these regions, thereby affecting transcript expression of nearby genes. Indeed, quantitative trait locus (QTL) analysis, a powerful approach for predicting the functional correlates of non-coding variants, has shown SNPs and CNVs to be associated with transcript expression^9–12^, and has proven to be useful in the interpretation of genome-wide association study (GWAS) results in complex traits.

Regulation of transcript expression is a complex process that is influenced by both underlying genetic and epigenetic mechanisms. An epigenetic mechanism that is well documented to influence transcript regulation is DNA methylation, which involves addition of a methyl group to cytosine residues within a CpG dinucleotide. Methylation of gene promoters is typically inversely correlated with transcript expression, whereas gene-body CpG hypermethylation has been reported to result in transcript overexpression^13^. Previous studies have reported that, similar to CNVs, DNA methylation patterns are variable both among different individuals (i.e., they are variably methylated regions, VMRs)^14^ and among different tissues within a given individual (i.e., they are tissue-specific differentially methylated regions, T-DMRs)^15^. In addition, monozygotic twins show epigenetic^16^ and genetic copy number^17^ variability. However, to date, virtually nothing is known about the relationship between germline CNVs and methylation patterns.

To address this question, in this study, we first tested whether inter-individual differences in DNA copy number is associated with inter-individual variation in DNA methylation levels by performing QTL analysis, using CNV genotype profiles from the 1000 Genomes Project^2^ and Conrad et al.^7^ studies, and paired DNA methylation data from Bell et al.^18^. We then evaluated whether the CNV-associated methylation changes were correlated with gene expression and whether CNVs, which were associated with methylation (CNV-mQTLs), were enriched for regulatory regions defined by the ENCODE Consortium and were involved in long-range physical interactions with the associated CpGs. In summary, we identified associations between specific genomic gains/losses and methylation of specific CpG islands in the same individuals. Our study establishes a relationship between CNVs and inter-individual DNA methylation patterns, and their impact on gene regulation.

## Results

### mQTL association analysis of CNVs with CpG methylation

We performed an association analysis between CNVs and CpG methylation using a discovery cohort of cell lines from 77 individuals—77 HapMap Yoruba in Ibadan, Nigeria (YRI) lymphoblastoid cell lines (LCLs)—with paired genome-wide CNV^2,7^ and methylation data^18^. Methylation data were obtained for a total of 19,254 CpG sites of 10,375 genes in the 77 individuals using the Illumina HumanMethylation27 DNA Analysis BeadChip assay^18^. For these 77 individuals, CNV genotypes were obtained from array CGH data^7^ (*n* = 4883) and also from next-generation sequencing data for a subset of 53 individuals (1000 Genomes Consortium^2^, *n* = 7240). Association between CNVs and CpG island methylation was examined using the QTL analysis method developed by Stranger et al.^10^. Briefly, Spearman’s rank correlation tests were performed on the CNV genotypes and CpG methylations. We then performed multi-test correction by randomly permuting the methylation of each CpG site 10,000 times^9,19^ and performing associations with the permutated dataset. A permutation threshold of 0.01 was used to define significant CNV-methylation associations or CNV-mQTLs.

We defined CNVs that lie within a 2 Mb window (1 Mb upstream and 1 Mb downstream of the midpoint) of any given CpG island as proximal associations (Supplementary Fig. 1). This large window size was chosen, because CNVs often span several kilobases and also have been previously reported to be involved in long-range gene regulation^9,20^. We defined CNVs that map beyond the 2 Mb window around a CpG island as distal associations.

**Fig. 1:**
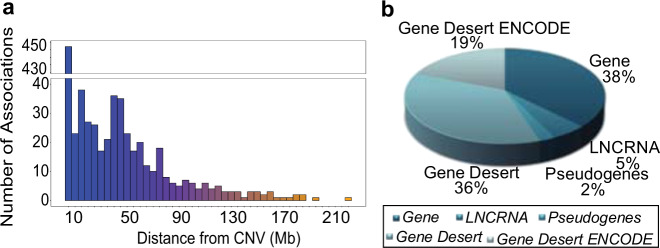
Methylation QTLs (mQTLs) and features. **a** The distance between the CNV-mQTLs and the associated CpG islands. **b** Distribution of CNV-mQTLs overlapping genic and non-gene (gene desert) regions.

We identified 851 (407 proximal and 444 distal) significant CNV-CpG associations (CNV-mQTLs) (Supplementary Fig. 2 and Supplementary Data 1), involving 656 unique CNVs that were correlated with the methylation of 738 CpG sites in 715 genes. Among these 851 CNV-mQTLs, 39 were associated with CpG methylation both proximally and distally. The overall permutation false discovery rate (FDR) was estimated to be <14% (FDR = [the number of genes tested × permutation *P-*value cutoff/the number of associated CpGs = 10,375 × 0.01/748 = 13.78%]). The distribution of CNV sizes for the significant CNV-mQTLs (size range = [51–426,206 bp]) identified did not differ from that of all the CNVs (size range = [50–1,102,849 bp]) (Kolmogorov–Smirnov test *P* = 0.2767) (Supplementary Table 1 and Supplementary Fig. 3). The vast majority of CNV-mQTLs (*n* = 567) had a minor allele frequency (MAF) > 5% (Supplementary Table 2). We did not observe any specific patterns of CNV-mQTLs across chromosomes. In addition, we did not find distal associations to be more subtelomeric CNVs (Supplementary Fig. 4a). To examine whether CNV-mQTLs were tagged by nearby SNPs, we performed linkage disequilibrium (LD) analysis between CNV-mQTLs (*n* = 656) and all bi-allelic SNPs^18,21,22^, which were located within 1 Mb of each other. We found that the vast majority of CNV-mQTLs (*n* = 555) were indeed in high LD with nearby SNPs (Pearson *r*^*2*^ > 0.5). Next, we assessed whether the CNV-mQTLs are in LD with published SNP-mQTLs (SNPs that are associated with CpG methylation) within 1 MB of the CNV-mQTLs. Our analysis showed that most of the CNV-mQTLs were in low LD with SNP-mQTLs, although these CNV-mQTLs may be tagged by nearby SNPs which are not known to be associated with CpG methylation (Supplementary Table 3). To further examine whether the CNV-mQTLs have an independent effect on CpG methylation compared with known SNP-mQTLs, we performed a conditional analysis of CNV-mQTLs and nearby known SNP-mQTLs. Results showed that 53.96% CNV-mQTLs (354 out of 656 CNV-mQTLs) have an independent genetic effect on the CpG methylation compared with nearby SNP-mQTLs (*p*-value < 0.05). Although 52.5% of the CNV-mQTLs were seen to be located within 5 Mb of their associated CpGs, long-range associations > 100 Mb were also observed (Fig. 1a and Supplementary Table 4). We observed a decreasing trend in frequency of associations with increasing distance. Forty-five percent of CNV-mQTLs overlapped genic regions (38% with genes, 5% with lncRNAs, and 2% with pseudogenes) and 55% with non-genic regions (Fig. 1b). The majority of CNV-mQTLs that contained genes were proximal CNV-mQTLs (Supplementary Fig. 4b). Gene-spanning CNV-mQTLs predominantly overlapped introns (Supplementary Fig. 4b).

**Fig. 2:**
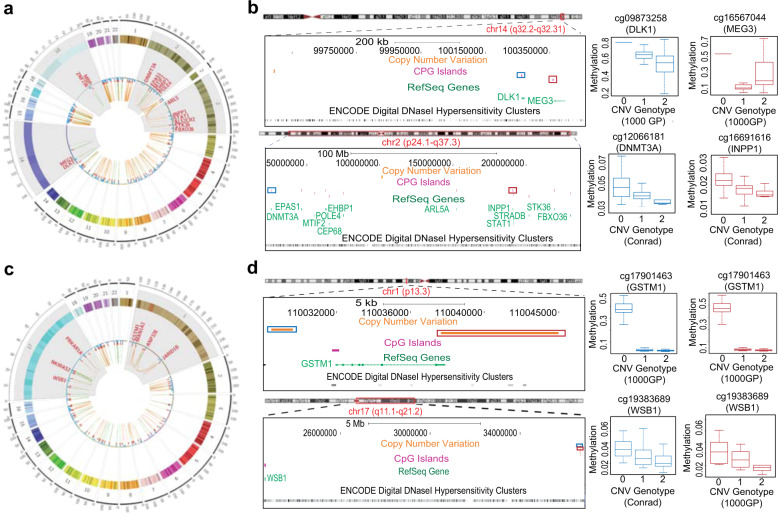
Types of mQTL associations. Shown are Circos plots of single CNVs associated with methylation of multiple CpG sites (**a**) and, conversely, multiple CNVs associated with methylation of a single CpG site (**c**). Chromosomes form the outer ring of the plot, followed by CpGs of genes (blue bars) and CNVs (dark orange bars). Arched lines indicate associations between CNVs and CpG methylation (proximal, green; distal, orange). Methylation of neighboring genes, *ZNF236* and *MBP*, located in the critical domain for 18q deletion syndrome, is associated with a common CNV-mQTL. **b** Upper panel (left): example of a CNV (orange), proximally associated with the methylation of CpG sites (boxed blue and red) in the promoters of the imprinted genes *DLK1* and *MEG3* (green). Lower panel (left): a single CNV-mQTL distally associated with multiple CpG sites in the promoters of *ALS2CR2*, *ARL5*, *CEP68*, *DNMT3A*, *EHBP1*, *EPAS1*, *FBXO36*, *INPP1*, *MTIF2*, *POLE4*, *STAT1*, and *STK36* genes. **d** Upper panel (left): example of multiple CNV-mQTLs proximally associated with a CpG site in the promoter of the gene *GSTM1*. Lower panel (left): multiple CNV-mQTLs distally associated with the methylation of a single CpG site in the promoter of the gene *WSB1*. Box and whisker plots on the right of **b** and **d** show correlation between CNV genotype and the associated CpG methylation.

We detected multiple instances where a single CNV was associated with multiple CpGs both proximally and distally (Fig. 2a, b). Among proximal associations, we saw that some single CNV-mQTLs were associated with methylation of neighboring genes. For example, *ZNF236* (Spearman’s *r*-value = −0.397) and the *MBP* (Spearman’s *r*-value = −0.307) genes, which both lie in the critical domain for 18q deletion syndrome^23^, are both associated with a common CNV-mQTL (Fig. 2a). Another example is the neighboring *DLK1* (Spearman’s *r*-value = −0.423), *MEG3* (Spearman’s *r*-value = 0.394) cluster, whose methylation patterns are associated with a common CNV-mQTL (Fig. 2b). The converse scenario, where multiple CNV-mQTLs were associated with the methylation of a single CpG island, was also observed (Fig. 2c, d). Examples include the *GSTM1* and the *WSB1* genes (Fig. 2d). Interestingly, we also found instances where methylation of groups of individual CpGs were all associated with multiple CNV-mQTLs. For example, three CNV-mQTLs on chromosome 1, which are in moderate and high LD (Pearson’s *r*^*2*^-values are 0.519, 0.416, and 0.828 for chr1:120557209–120738188 vs. chr1:142612557–142700497, chr1:120557209–120738188 vs. chr1:204239359–204366782, and chr1:142612557–142700497 vs. chr1:204239359–204366782, respectively), were all distally associated with the CpG methylation patterns of three different genes: *JARID1B*, *MAN1A2*, and *ANP32E*. The presence of such associations underscores a strong correlation between methylation of these genes with genetic variation at the above-mentioned loci. Lastly, CNV-mQTLs were also seen to be associated with CpG methylation of several imprinted genes (Supplementary Table 5).

### Comparison with differentially methylated regions in the genome

We compared CNV-mQTL-associated CpGs with published VMRs^14^ and T-DMRs (14), to determine how much of the methylation variation is associated with genetic variation. We found that 3.1% (7/227) of previously reported VMRs and 3.6% (592/16,379) of previously reported T-DMRs overlap CNV-mQTL-associated CpG islands and shores. Next, as differentially methylated regions have been shown to be associated with SNPs^24^, we compiled CNV-mQTL-associated CpGs from our study with CpGs associated with published SNP-mQTLs^18,21,25^ and examined whether they have been previously reported to be VMRs or T-DMRs (see “Methods”). We found that 28.6% (65/227) VMRs and 27.7% (4,533/16,379) T-DMRs overlap with CpG islands and shores associated with CNV- and SNP-mQTLs. This shows that some amount of differential methylation is associated with genetic variation.

### Validation of CNV-associated methylation regions

We performed pyrosequencing to validate the methylation levels as assessed by the arrays in the discovery set and then confirmed the correlations with copy number. Towards this, 30 loci that showed a significant association with CNV-mQTLs were selected and 27 of these, for which primers (Supplementary Table 6) could be designed, were queried. Pyrosequencing showed a concordance of 77.8% considering all the copy number states (Supplementary Fig. 5).

Further, we generated methylation data from 24 HapMap individuals (Supplementary Table 7), including one HapMap trio with European ancestry in Utah (CEU), and one HapMap YRI trio that were both sequenced by the 1000 Genomes Consortium. Prior to association analysis, principal component analysis was performed on the genotype and methylation data (*β*-values) from the 24 samples to confirm the absence of stratification (Supplementary Fig. 6). Following this confirmation, we obtained CNV genotypes for these individuals from the 1000 Genomes^4^ and HapMap^7^ studies. This yielded 496 CNVs that were identified in the discovery dataset and were copy number variable in the validation dataset. Using the same pipeline for identifying CNV-mQTLs in the discovery set, we found that 216 (43.5%, *P* < 0.01) and 344 (69.4%, *P* < 0.05) of the CNV-mQTLs identified in our discovery set were also associated with CpG methylation in the validation set (Supplementary Data 2).

### Association of CNVs with methylation and gene expression

**Fig. 3:**
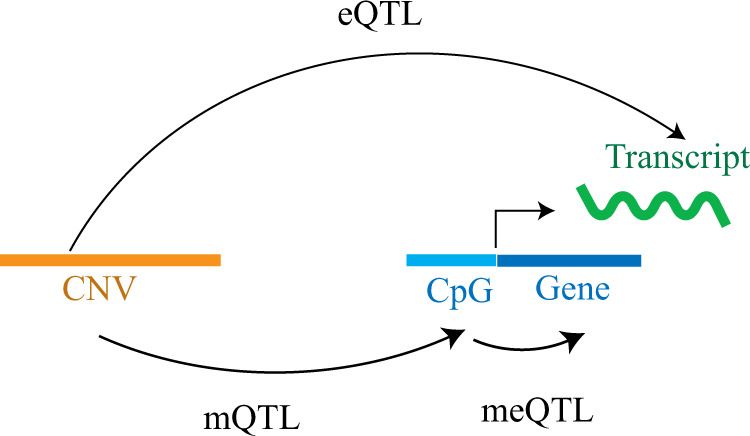
Summary of association with gene expression. Schematic representation of eQTL (CNV associated with expression), mQTL (CNV associated with methylation of CpG), and eQTM (association of an mQTL-associated-CpG with expression).

Next, we examined the expression to methylation associations (methylation-expression QTLs, eQTMs) by considering only those CpGs whose methylation was significantly correlated with CNV-mQTLs (eQTM, Fig. 3). RNA-sequencing derived estimates of gene expression levels in LCLs were obtained for 69 HapMap YRI individuals^26^. We defined the boundaries for proximal and distal interactions using the midpoint of an mQTL-associated CpG site as earlier (Supplementary Fig. 1). Using Spearman’s rank correlation and permutation tests, we identified 242 eQTM significant associations (permutation test, *P* < 0.01) representing 225 RefSeq genes (83 proximal and 142 distal RefSeq genes) (Supplementary Data 3). Among these 242 eQTM associations, 116 were positive correlations, whereas 126 were negative correlations. Although methylation is typically negatively correlated with gene expression, similar patterns of positive and negative correlations, at the population level, have been reported by several studies^27,28^. An alternate modification of DNA methylation, involving 5-Hydroxymethylcytosine (5hMc), has been hypothesized to underlie this observed positive correlation, as 5hMc levels are positively correlated with gene expression^25^. Supplementary Fig. 7 depicts representative eQTMs for proximal and distal associations.

Next, we sought to evaluate whether CNVs act as both methylation QTLs (mQTLs) and expression QTLs (eQTLs). The presence of such CNVs would suggest a possible mechanism by which CNVs may influence transcript expression. Towards this, we first identified CNV eQTLs in our dataset (Supplementary Data 4) and then looked for common CNVs that were also present in the CNV-mQTL dataset. This analysis yielded 79 common CNVs that were both eQTLs and mQTLs, associated with the expression of 81 genes and methylation of 89 genes, respectively. Among these common CNVs, ten were associated with methylation and expression of the same genes showing that some of the effect of CNVs on expression could be mediated through mQTL-associated differential methylation of CpG sites in the promoters of these genes. In addition, the expression of 21 genes was associated with CNV eQTLs and mQTLs (Supplementary Data 5). Hence, in our dataset, separate CNVs act as either eQTLs or as mQTLs. Our observations agree with previous reports where separate SNPs were associated with expression and methylation^28^. Therefore, if a genetic variant plays a causal role in regulation of expression and methylation, our data suggest that the underlying mechanism is complex where independent CNVs modulate gene expression and methylation, which in turn influence each other.

### Overlap with ENCODE regulatory sequences

**Fig. 4:**
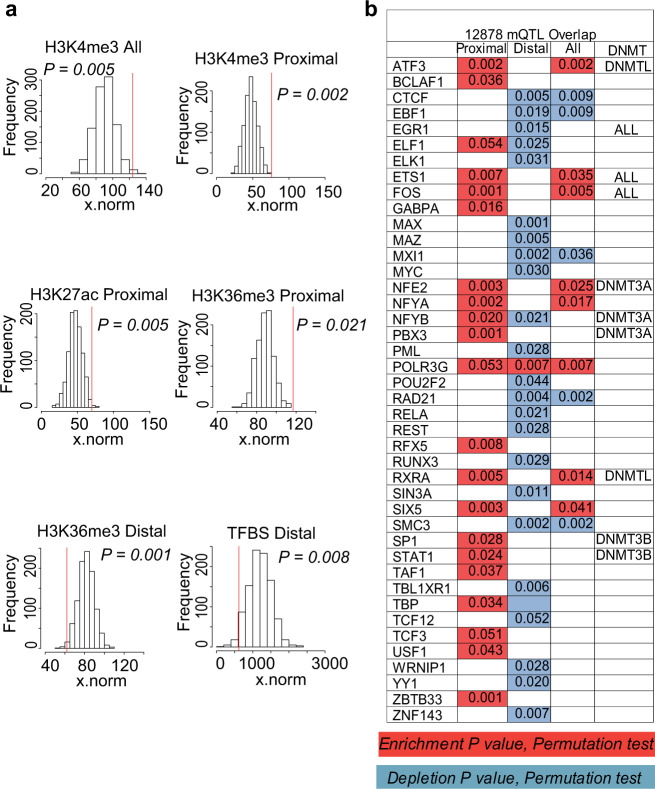
ENCODE analysis. Significance testing of the overlap between CNV-mQTLs and various histone modifications, and TFBS defined by the ENCODE consortium on the HapMap cell line GM12878. **a** Null distribution of overlap between randomly permutated datasets (1000 iterations) and ENCODE features. The red line shows the statistical significance of the overlap of the CNV-mQTL dataset. *P*-values from permutation test are shown. **b** Table summarizes the permutation test *P*-values of overlap between proximal/distal/all CNV-mQTLs and individual TFBS. Enrichment and depletion are indicated in red and blue, respectively. The last column summarizes the various DNA methyl transferases (DNMTs) that have been demonstrated to interact with each TF^61^.

We further sought to determine whether CNV-mQTLs overlap regulatory sequences, to understand the potential mechanisms by which CNVs affect methylation. The data generated by the ENCODE consortium^8^ on the GM12878 cell line were utilized for this analysis. We first determined direct overlaps between mQTLs and multiple ENCODE features including DNase hypersensitivity sites, regulatory sites marked by histone modifications, namely H3K4me3 (promoters), H3K4me1 (poised enhancers), H3K27Ac (active enhancers), and H3K36Me3 (marks 3′-end of active genes), as well as TFBS. We determined that 44% of our identified CNV-mQTLs overlapped ENCODE regulatory marks (Supplementary Fig. 8). Of the “gene desert” CNV-mQTLs that did not overlap with genes, 35% contained regulatory regions as defined by ENCODE (Fig. 1b). Following a direct overlap, random permutation analysis was performed to estimate the statistical significance of the observed overlap. Here, a null distribution was estimated from overlaps with 1000 randomized permutations of chromosomal regions with the same number and size distribution as our CNV-mQTLs. This analysis showed that H3K4Me3 promoter mark was enriched among all CNV-mQTLs (permutation test, *P* = 0.005). In addition, proximal mQTLs were significantly enriched for enhancer H3K4Me1 marks (permutation test, *P* = 0.002) and the H3K27Ac active enhancer sequences (permutation test, *P* = 0.02) (Fig. 4a).

We also observed an enrichment of H3K36Me3 mark in proximal mQTLs (permutation test, *P* = 0.001), whereas distal mQTLs were depleted for the same H3K36Me3 mark (permutation test, *P* = 0.008). Similarly, proximal mQTLs were enriched for specific TFBS, whereas distal mQTLs were depleted for TFBS (permutation test, *P* = 0.017), (Fig. 4a, b and Supplementary Fig. 9). The only site that was enriched among all CNV-mQTLs was the Pol3-binding site (Fig. 4b). Our analysis therefore demonstrated that CNV-mQTLs are indeed enriched for regulatory sequences.

### Long-range interactions of CNVs and associated CpGs

**Fig. 5:**
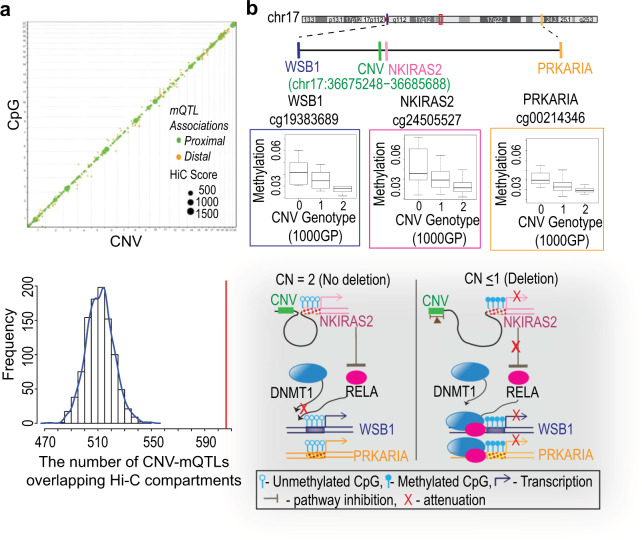
Long-range interactions. **a** Top: whole-genome summary of CNV-mQTL/CpG pairs that overlap with interacting Hi-C regions. The size of each point corresponds to the Hi-C interaction signal, denoted by the KR normalization score (green, proximal CNV-mQTLs; orange, distal CNV-mQTLs). **a** Bottom: enrichment analysis. The bars show the frequency of permutated CNV-mQTL/CpG datasets overlapping with Hi-C compartments at 10 kb resolution; the blue fitted line shows the distribution of 1000 permutation sets; the red line denotes the number of actual CNV-mQTL/CpG pairs overlapping with Hi-C compartments (*p* < 0.001, permutation test). **b** Schematic of a potential mechanism by which CNV-mQTLs may influence distal CpG methylation. Ideogram shows three CNV-mQTLs (red boxed region) that are all associated with the methylation of the genes *NKIRAS2* (proximal*)*, *WSB1* (distal), and *PRKAR1A* (distal). Box and whiskers plots show correlation between the mQTL and each of the three genes. *NKIRAS2* inhibits the activation of the *RELA* subunit of the TF, NFκB. NFκB is known to regulate *WSB1* methylation through its interaction with *DNMT1*. In a normal state, looping interactions between the CNV-mQTLs and the proximally associated gene *NKIRAS2* could potentially activate its expression and inhibit NFκB activation, thereby decreasing methylation *WSB1* and *PRKAR1A*. Conversely, a deletion in the CNV-mQTL region may lead to reduced *NKIRAS2* expression and, thereby, increased methylation of the distally associated genes, *WSB1* and *PRKAR1A*, mediated by activated NFκB.

We analyzed whether CNV-mQTLs are capable of physically interacting with the associated CpG islands by using data from the high-throughput chromosome conformation capture (Hi-C) approach that maps long-range interactions in genome. Publicly available Hi-C data from the HapMap cell line GM12878^29^ were used to test for overlap between interacting regions mapped by Hi-C and CNV-mQTL/CpG pairs from the discovery cohort (see “Methods”). We found that a total of 606 CNV-mQTLs and associated CpGs (with a 5 kb window around the CpG island to include shore regions) overlapped with 10 kb resolution Hi-C compartment dataset. We observed that proximal CNV-mQTL/CpG pairs have higher Hi-C interactions compared with distal CNV-mQTL/CpG pairs (Fig. 5a and Supplementary Fig. 10). The number of overlaps at different Hi-C resolutions and window sizes around the CpG island are summarized in Supplementary Data 6–9. This analysis suggests that CNV-mQTLs physically interact with the islands and shores of the associated CpGs. Next, we conducted permutation test to assess whether CNV-mQTL/CpG pairs were statistically enriched for Hi-C interactions. We generated 1000 random datasets for the two interacting regions that followed the same size and distance distributions as the CNV-mQTL regions and the associated CpG shore regions on each chromosome (see Supplementary Methods). This analysis showed that CNV-mQTLs and associated CpGs were enriched for Hi-C interactions (permutation test, *P* < 0.001; Fig. 5a and Supplementary Fig. 10).

### Disease-associated variants

GWAS have revealed the potential role of genetic variants in conferring risk to various diseases. Recent reports have shown that loss-of-function genetic variants in healthy genomes overlap open reading frames of genes^30^. In a similar context, we examined whether CNV-mQTLs or associated genes were previously reported by GWAS. Indeed, we found that some of the CNVs previously shown to be associated with disease risk were mQTLs in our dataset (Supplementary Data 10). In addition, genes previously shown to confer disease risk were associated with CNV-mQTLs. This includes the *SNCA* gene whose increased copy number has been established to be associated with Parkinson’s disease. Interestingly, studies have shown that hypomethylation of a CpG site in the promoter of *SNCA* gene is associated with increased risk to Parkinson’s disease^31^.

Next, as a larger number of GWAS have been carried out using SNPs, we asked whether the genes identified by SNP GWAS studies are associated with CNV-mQTLs. The SNPs for this analysis were derived from the GWAS catalog (www.ebi.ac.uk/gwas, version v1.0, accessed 30 March 2015). We observed that 230 genes that were identified by SNP association studies were also associated with CNV-mQTLs in our study (Supplementary Data 11). One interesting observation was that, genes that are associated with a common SNP risk variant are also associated with a common CNV-mQTL. An example includes the genes *MPHOSPH1* and *CH25H*, both associated with a common CNV-mQTL (proximal and distal, respectively) and reported to be associated with a common SNP conferring risk to Alzheimer’s disease^32^. Interestingly, the CNV-mQTL is in low LD with the reported SNPs. In addition, we found an instance where a common CNV-mQTL is associated with the methylation of *CUTL2 (CUX2)* and *FAM109A* genes that lie in the 12q24 LD, and reported to confer risk to type I diabetes^33^. Finally, CNV-mQTLs were found to be associated with CpG island methylation of genes frequently altered in cancer, e.g., *PTEN*, *RB1*, *ERBB2*, *WNT1*, *WNT4*, *WNT11*, *MAPK15*, and *MAPK6*. These data show that identifying the genes associated with CNV-mQTLs may lead to a better understanding and interpretation of GWAS data. Indeed, association studies are now being focused on identifying epigenetic variants linked with diseases^34^.

## Discussion

We demonstrate, in this study, that germline inter-individual CNVs are correlated with epigenetic variability in the human genome. Further, we show that the associated mQTL-CpG patterns are correlated with transcript expression, are enriched for regulatory features, are involved in long-range interactions, and are among previously reported disease risk loci. The dataset in this study mainly allowed for discovery of associations with common CNVs (MAF > 5%), although some associations with low-frequency CNVs (MAF < 1%) were also represented. The FDR is consistent with previous studies^9,10^. Genetic influence on methylation has now been described by several studies that have demonstrated SNPs to be associated with DNA methylation, both at an inter-individual and a population-scale level^18,21,25,35–37^. A recent study by Sun et al.^38^ demonstrated that somatic copy number alterations in cancer are associated with DNA methylation. Multiple studies suggest that genetic variation may have a causal role in regulating CpG methylation^25,28^. Conversely, it is also possible that methylation could lead to CNV formation as demonstrated by studies showing that methylation leads to increase in DNA breakage^39^. Our data show that CNV-mQTLs are in low LD with known SNP-mQTLs located within 1 MB of CNV, whereas CNV-mQTLs are in high LD with nearby SNPs (within 1 MB of CNV) that are not known to be associated with CpG methylation. We further performed conditional analysis between CNV-mQTLs and nearby known SNP-mQTLs, and showed that over half of identified CNV-mQTLs have an independent genetic effect on methylation when considering nearby SNP-mQTLs. We expect that future studies with larger sample sizes and better characterization of CNVs will allow for joint association studies that provide further insights into potential causal effects of SNPs and CNVs on CpG methylation. We believe that the limited overlap observed with differentially methylated regions in the genome would be an underestimate, as VMRs, including CpG island shores^14^, are not interrogated by the methylation platform used in this study.

The fact that multiple CNVs are associated with the methylation of a single CpG and a set of CNV-mQTLs are associated with a group of CpGs indicate a potentially strong genetic control and plasticity of methylation states. One intriguing observation made in our study was that CNV-mQTLs were associated with methylation patterns of 12 imprinted genes. Imprinted genes have been previously seen to have an allele-specific methylation pattern^17,35^. As imprinting patterns are established during development, we hypothesize that CNVs and SNPs may act to “fine-tune” methylation of imprinted genes. Our observation that separate CNVs are associated with expression and methylation is similar to the observations made with SNPs in LCLs where methylation and expression are independently affected by SNPs^28^. Our data further showed that CNV-mQTLs are associated with CpG methylation of genes identified by GWAS studies and in cancer. CNVs being associated with neighboring genes may have implications on cancer studies, where alteration of methylation patterns of adjacent genes is often reported.

Studies have demonstrated that CpG island methylation is regulated by sequences in *cis*, showing that the sequence content around CpGs plays a key role in determining the pattern of CpG methylation^40,41^. The enrichment of enhancer sites among CNV-mQTLs suggests a regulatory role. Our results agree with recent studies that have shown concordant changes of methylation associated with other chromatin features such as histone modifications^25,42,43^. In addition, investigations have shown that binding of transcription factors influences DNA methylation^40,41,44^. Specifically, transcription factors have been shown to interact with DNA methyl transferases (DNMTs) and recruit them for targeted methylation^40,45,46^. Studies have also indicated that SNPs, by overlapping TFBS, can potentially affect DNA methylation^25,28^. In our study, many of the TFs that were enriched in proximal mQTLs have been shown by earlier studies to bind to DNMTs (Fig. 4b and Supplementary Fig. 9). For example, SP1and ELF1 have previously been shown to either regulate or are predictive of methylation, respectively^22,47^. Therefore, binding of TFs to CNV-mQTLs in promoters or enhancer sequences may potentially influence DNA methylation of the associated CpGs by long-range interactions. Our analysis of Hi-C data showed that a subset of CNV-mQTL/CpG pairs are indeed in regions that show long-range interactions. While our observations indicate a potential mechanism by which proximal CNVs may regulate methylation, the effects of distal mQTLs may be mediated through the proximal associations by influencing the expression of piRNAs or genes that can regulate methylation of distal genes. This concept has been also suggested by SNP-mQTL studies^48,49^. An example of one such indirect interaction in our dataset involves three CNV-mQTLs that were each associated with the same set of three genes, *NKIRAS2*, *WSB1*, and *PRKR1A*. This showed a strong genetic association with CpG methylation of these genes. While *NIKIRAS2* was associated proximally, the other two genes were distally associated. NKIRAS2 is known to inhibit the activation of the transcription factor RELA^50^. ENCODE data showed RELA-binding sites in the promoter of both the distally associated genes. RELA has been shown to interact and recruit DNMT1 to CpG islands^51^. Therefore, our findings suggest the manner by which the CNV-mQTL may influence the methylation and subsequent expression of *NKIRAS2* proximally, thereby modulating RELA mediated distal methylation (Fig. 5b). Although this suggests a potential mechanism of how CNVs may influence DNA methylation, more studies are required to establish a causal role and rule out a simple correlation. Finally, we see depletion in CTCF sites possibly owing to the recent observation that the binding sites are “buffered by genetic and epigenetic perturbance”^52^.

Despite the presence of widespread germline copy number variants in the genome, their effects on cellular phenotype, when compared to SNPs, is less understood. The significance of this study lies in demonstrating that inter-individual germline CNVs are associated with variation in CpG island methylation in the genome, are enriched for regulatory sequences, including transcription factor-binding sites, and are able to engage in physical interactions with the associated CpG. The observations being presented here, substantially enhance our growing understanding of the relationship between genetic and epigenetic variation in the genome. Our findings have broad implications on understanding the effects of structural variation on cellular phenotype, specifically on the fundamental mechanisms of gene regulation, as well as in complex traits underlying evolution and disease.

## Methods

### CNV data

We utilized two published high-resolution datasets by Conrad et al.^7^ and the population-scale whole-genome sequencing data released by the 1000 Genomes Project^2^ for this study. For the methylation association analysis, we included 7240 autosomal CNVs whose genotypes vary in 53 YRI individuals sequenced at low-depth as a part of the 1000 Genomes Project and 4883 autosomal CNVs that have variable genotypes in the 77 YRI individuals by Conrad et al.^7^.

### Methylation data

We downloaded methylation profiles of 77 YRI individuals generated using the Illumina HumanMethylation27 Beadchip assay, as published in the Bell et al.^18^ study. This array includes 27,578 probes that target the CpG sites located near the transcription start sites of genes. We extracted methylation profiles for the 53 individuals who have genotyped CNV data in the 1000 Genomes dataset^2^ and the 77 individuals who have been genotyped by Conrad et al.^7^ analysis.

### Genome-wide CNV-methylation association analyses

We performed two sets of CNV-methylation association analyses, which focused on (i) the 1000 Genomes Project CNV genotypes from 53 YRI individuals^2^ and (ii) the Conrad et al.^7^ CNV genotypes from 77 YRI individuals. Methylation profiles of 19,254 CpG sites of genes (with genomic coordinate information) from the same individuals were used for the analysis. Association analysis was performed independently in each of these two datasets. We first conducted Spearman’s rank correlation for CNVs within 1 Mb upstream and downstream of the midpoint of a CpG site for any given gene (proximal associations). We also computed correlation of gene methylation with probe ratios of all CNVs on the same chromosome as the gene but beyond the 2 Mb window around the CpG site (distal associations). *r*-values shown represent the correlation between CNVs and CpGs. Negative *r*-values represent anti-correlation, whereas positive values indicate direct correlation. Permutation-based multiple-test correction, which involves random permutation of the methylation phenotypes 10,000 times, was then applied to both proximal and distal association analysis. To call association for CNV-methylation pairs significant, we considered a permutation *P*-value cutoff of 0.01.

### Linkage disequilibrium analysis

We tested for LD between CNV-mQTLs and all nearby SNPs, as well as published SNP-mQTLs^18,21,25^, using bi-allelic SNP genotypes and CNV genotypes in matched samples from the YRI population. The bi-allelic SNP genotypes were extracted from the 1000 Genomes Project, phase 3 release^3^. Prior to LD analysis we first performed liftover, to convert the genomic coordinates of SNPs to hg18 from hg19, using the CrossMap^53^. We then used bedtools -window^54^ to select SNP-mQTLs that were located within 1 Mb window of CNV-mQTLs to calculate LD. Pearson’s correlation computed using Python 1.17.2 (*numpy*), was used to calculate the LD between a CNV-mQTL and SNP under investigation. The *r*^*2*^ values denote the correlation between CNV-mQTLs and SNPs. In instances where multiple SNPs were located near a CNV-mQTL, we only considered the SNP with the largest *r*^*2*^-value for reporting.

### Conditional analysis between CNV-mQTLs and known SNP-mQTLs

To evaluate whether CNV-mQTLs have an independent effect on CpG methylation compared with known SNP-mQTLs, we conducted a conditional analysis^55,56^ of our CNV-mQTLs with known SNP-mQTLs^18,21,25^. This conditional analysis will estimate if CNV-mQTLs are still main genetic factors affecting CpG methylation when nearby SNP-mQTLs are taken into account. For each CNV-mQTL, we evaluated all the known SNP-mQTLs^18,21,25^ within 1 Mb window upstream and downstream of the CNV-mQTL. For all nearby known SNP-mQTLs, we extracted genotypes from the 1000 Genomes Project phase 3 release^3^. We then re-evaluated the association between each CNV-mQTL and CpG, with this association being conditional on nearby known SNP-mQTLs using cpgen implemented in an R package (https://github.com/cheuerde/cpgen)^57–59^. The threshold value used for conditional analysis was *p* < 0.05 and CNV-mQTLs that passed this threshold were considered to have an independent effect on CpG methylation when compared with nearby known SNP-mQTLs.

### Comparison with VMRs and T-DMRs

The comparison with known VMRs^14^ and T-DMRs^15^ was determined by doing 1 bp intersection of VMRs/T-DMRs with the CpG islands tagged by the CNV-/SNP-associated CpGs. We also included the region around the CpG islands, the CpG island shores, to perform the overlap, as they have been reported to be differentially methylated. CpG island shores were defined as 2 kb upstream and downstream of the CpG islands as proposed by Irizarry et al.^15^.

### Methylation-expression QTL analysis

#### Expression data

Estimates of gene expression levels were obtained from Pickrell et al.^26^, which includes the RNA-sequencing data for 12,028 genes from 69 YRI individuals. Seven hundred and forty-eight CpG sites, which were found to be associated with CNV-mQTLs, were included in the analysis.

#### Association analysis

We examined the correlation between methylation profiles of CpG sites within the 2 Mb neighborhood of the target gene and the expression of that target gene (proximal associations). Distal association analysis was carried out by examining correlation of methylation of CpG sites outside the 2 Mb window but on the same chromosome. A 10,000 permutations test was performed as earlier and a permutation *P*-value cutoff of 0.01 was used to select eQTM associations.

### CNV-expression QTL analysis

CNV genotypes from YRI individuals from the 1000 Genomes^2^ (*n* = 53) and Conrad et al.^7^ (*n* = 77) datasets were each used to perform CNV-expression association analysis with the expression profiles of 69 YRI individuals^26^. By extracting data for individuals with both varied genotypes and expression profiles, we obtained 7172 CNVs for 49 individuals in the 1000 Genomes dataset^2^ and 3929 CNVs for 67 individuals in the Conrad et al.^7^ dataset. For each of the two datasets, we performed association analyses with the expression profiles of 12,028 genes in the corresponding individuals. Proximal association was examined by checking the correlation between the CNV genotypes within the 2 Mb neighborhood of the target gene and the expression profiles of the target gene. Distal association analysis was performed in a similar manner but considering CNVs outside the 2 Mb window but on the same chromosome of targeted genes. A 10,000 permutations test was performed and a permutation *P*-value cutoff of 0.01 was used to identify CNV-eQTL associations. The list of CNV eQTLs was then compared to CNV-mQTLs, to identify common CNVs.

### CNV-mQTL validation

#### Cell lines and DNA extraction

The HapMap cell lines were purchased from Coriell Institute and were maintained in RMPI 1640 (Life Technologies) supplemented with 10% fetal bovine serum (Atlanta Biologicals). DNA was extracted from cell lines using the DNeasy Blood and Tissue kit (Qiagen) and was used for the PCR analysis.

#### Pyrosequencing

Two micrograms of DNA from the entire Yoruba population panel was obtained from Coriell Cell Repository at a concentration of 100 ng/µl. One microgram of DNA from each selected individual was then subjected to bisulfite conversion using the Epitect Bisulfite Kit (Qiagen) as per the manufacturer’s protocol. PCR and sequencing primers were designed for the selected regions using the PyroMark Assay Design SW 2.0 (Qiagen) and custom Pyromark® CpG assays (Qiagen) were ordered (Supplementary Table 6). One of the two PCR primers is biotinylated. PCR was then performed on the bisulfite converted samples using the PCR primers from the custom assay kit to differentiate methylated cytosine (mC) from unmethyated cytosine (C). The PCR product was subsequently purified using the MinElute PCR purification kit (Qiagen). The purified PCR product was checked for quality on a 1.5% gel and then analyzed by Pyrosequencing. PyroMark Q24 software was used to analyze the results. Results were presented as methylation percentage values for each CpG in target region of the analyzed samples. The methylation percentage was calculated as an average of the methylation values of each CpG in the target region.

#### Illumina methylation 27 array analysis

We performed Illumina methylation 27 array (Illumina) analysis on 24 HapMap individuals including 1 CEU trio (family 1463 including NA12878, NA12891, NA12892) and 1 YRI trio (family Y117 including NA19240, NA19238, NA19239) (Supplementary Table 7). Of these 24 individuals, 15 were genotyped individuals in the 1000 Genomes Project^2^ and all the 24 individuals were genotyped in Conrad et al.^7^. Following preprocessing, similar to the strategy used in Bell et al.^18^, the *β*-values that capture methylation levels of probes on the array were quantile-normalized and applied for association studies. As in the discovery analysis, we performed CNV-methylation association analyses for the two CNV datasets: CNV genotypes reported in the 1000 Genomes Project^2^ and CNV genotypes from Conrad et al.^7^. Of these associations, only CNV-mQTLs from the discovery dataset that were copy number variable in the validation dataset were considered using a *P*-value cutoff of < 0.05.

### Imprinted gene analysis

The list of imprinted genes were compiled from Morison et al.^60^ and the Geneimprint website (http://www.geneimprint.com/).

### ENCODE analysis

The data from the ENCODE consortium^8^ for DNase I hypersensitivity sites, transcription factor-binding sites and the ChipSeq data for the histone marks (H3K4Me1, H3K4Me3, H3K27Ac, and H3K36Me3) from the HapMap cell line GM12878, were downloaded from UCSC genome browser. The narrow peaks from the University of Washington DNase I dataset, was used for the analysis. The broad peaks from the Broad Institute dataset were downloaded for the histone modifications queried. The details are presented in Supplementary Methods.

### Assessing long-range interactions between CNVs and associated CpGs using Hi-C

More than 4.9 billion pairwise Hi-C contacts, at different resolutions (10 kb and 5 kb resolutions) across entire 22 autosomal, were obtained from in situ Hi-C analysis of the GM12878 cell line^29^. The coordinates of Hi-C compartments were first lifted back from b37 to b36, to match the CNV-mQTL coordinates. Then, the CNV-mQTL regions and their associated CpG regions (with an extended window size of 2 and 5 kb away from the CpG Island to include shores) were intersected with Hi-C compartments at 1 bp level using Bedtools intersect^54^. Finally, the interaction signal of each overlapped Hi-C compartment was calculated for CNV-mQTL based on the corresponding KRnorm values^29^.

### Permutation test for Hi-C enrichment analysis

We performed random permutation tests to see whether CNV-mQTL/CpG pairs were enriched for Hi-C interaction signals. Towards this, 1000 permutation datasets were randomly generated using Bedtools shuffle^54^, that followed the same distributions of CNV-mQTL regions and their associated CpG regions (with extended window size of 5 kb to include shores around from the CpG Island). All permutation datasets were generated as in the following three steps—first, random CNV regions that had the same size distribution as the CNV-mQTLs were randomly picked from each chromosome; second, random CpG regions that had the same size distribution as the associated CpG shore regions were randomly picked on the same chromosome; third, the random CNV regions and random CpG regions were randomly paired together with distances matching the distances between CNV-mQTLs and the associated CpGs. Next, we intersected the random CNV regions and randomly paired CpG regions with Hi-C compartments generated at 10 kb resolution. The number of random CNV-CpG pairs that overlapped with Hi-C compartments with non-zero signals across entire 22 autosomal chromosomes were counted for each of the 1000 permutated datasets. Finally, the log ratios were calculated using the number of real CNV-mQTL/CpG pairs overlapping with Hi-C 10 kb compartments against the permutated sets of CNV-CpG pairs overlapping with Hi-C compartments.

### Reporting summary

Further information on research design is available in the Nature Research Reporting Summary linked to this article.

## Supplementary information

Supplementary Information

Reporting Summary

Supplementary Data 1

Supplementary Data 2

Supplementary Data 3

Supplementary Data 4

Supplementary Data 5

Supplementary Data 6

Supplementary Data 7

Supplementary Data 8

Supplementary Data 9

Supplementary Data 10

Supplementary Data 11

## Data Availability

The DNA methylation dataset, generated for validation studies, supporting the conclusions of this article is available in the Gene Expression Omnibus (GEO) repository (https://www.ncbi.nlm.nih.gov/geo), accession number: GSE114131. Additional data are presented in the Supplementary Information section.

## Code availability

The code used in the paper can be accessed at https://github.com/ssetlur/setlur-lab/tree/master/CNVmQTL and https://github.com/shilab/CNVmQTL/.
