# Supplementary material for: Association of CNVs with methylation variation
Source: NPJ Genom Med. 2020 Sep 24;5:41. doi: 10.1038/s41525-020-00145-w (PMC7519119; doi:10.1038/s41525-020-00145-w)
Supplement: Supplementary file 1 — Supplementary Information [file 41525_2020_145_MOESM1_ESM.docx]

**Supplementary Information**

**Association of CNVs with methylation variation**

Xinghua Shi^1#^, Saranya Radhakrishnan^2^, Jia Wen^1^, Jin Yun Chen^2^, Junjie Chen^1#^, Brianna Ashlyn Lam^1^, Ryan E. Mills^3^, Barbara E. Stranger^4^, Charles Lee^5*^and Sunita R. Setlur^2*^

Sunita R. Setlur, Charles Lee

Email: ssetlur@rics.bwh.harvard.edu (SRS) and Charles.Lee@jax.org (CL)

**This PDF file includes:**

Contents

[**Supplemental Methods** 2](#_Toc45571244)

[**Fig. S1.** 5](#_Toc45571245)

[**Fig. S2.** 6](#_Toc45571246)

[**Fig. S3.** 7](#_Toc45571247)

[**Fig. S4.** 8](#_Toc45571248)

[**Fig. S5.** 10](#_Toc45571249)

[**Fig. S6.** 11](#_Toc45571250)

[**Fig. S7.** 12](#_Toc45571251)

[**Fig. S8.** 13](#_Toc45571252)

[**Fig. S9.** 18](#_Toc45571253)

[**Fig. S10.** 19](#_Toc45571254)

[**Table S1. Distribution of CNV-mQTL lengths** 20](#_Toc45571255)

[**Table S2. Statistics of the MAFs of CNV-mQTLs** 20](#_Toc45571256)

[**Table S3. Statistics of the LDs (r^2^) between CNV-mQTLs and nearby SNPs / SNP-mQTLs** 20](#_Toc45571257)

[**Table S4. The distance between CNV-mQTLs and CpGs** 20](#_Toc45571258)

[**Table S5. CNV-mQTLs associated with methylation of imprinted genes.** 21](#_Toc45571259)

[**Table S6. Primers** 22](#_Toc45571260)

[**Table S7. Validation Samples** 23](#_Toc45571261)

**Additional Supplementary Data sets uploaded as separate files:**

Supplementary Data 1: CNV methylation QTLs

Supplementary Data 2: CNV-mQTL Validation

Supplementary Data 3: Methylation Expression QTLs (eQTMs)

Supplementary Data 4: CNV-eQTL

Supplementary Data 5: Overlap between CNV-eQTLs and CNV-mQTLs

Supplementary Data 6: CpG_CNV_10kb_5kb: Hi-C region overlap with CNV-mQTL/ CpG pairs (10 kb

Hi-C resolution, 5 kb CpG Window)

Supplementary Data 7: CpG_CNV_10kb_2kb: Hi-C region overlap with CNV-mQTL/ CpG pairs (10 kb

Hi-C resolution, 2 kb CpG Window)

Supplementary Data 8: CpG_CNV_5kb_5kb: Hi-C region overlap with CNV-mQTL/ CpG pairs (5 kb Hi-

C resolution, 5 kb CpG Window)

Supplementary Data 9: CpG_CNV_5kb_2kb: Hi-C region overlap with CNV-mQTL/ CpG pairs (5 kb Hi-

C resolution, 2 kb CpG Window)

Supplementary Data 10: CNV-mQTLs reported in GWAS studies

Supplementary Data 11: Genes reported in SNP association studies

# **Supplemental Methods**

**ENCODE Overlap analysis**

The URLs used for the analysis are as below -

[wgEncodeUwDnaseSeqPeaksRep1Gm12878.narrowPeak.gz](http://hgdownload.cse.ucsc.edu/goldenPath/hg18/encodeDCC/wgEncodeUwDnaseSeq/wgEncodeUwDnaseSeqPeaksRep1Gm12878.narrowPeak.gz)

<ftp://hgdownload.cse.ucsc.edu/goldenPath/hg18/encodeDCC/wgEncodeBroadChipSeq/wgEncodeBroadChipSeqPeaksGm12878H3k27ac.broadPeak.gz>
<ftp://hgdownload.cse.ucsc.edu/goldenPath/hg18/encodeDCC/wgEncodeBroadChipSeq/wgEncodeBroadChipSeqPeaksGm12878H3k4me1.broadPeak.gz>

<http://hgdownload.cse.ucsc.edu/goldenPath/hg18/encodeDCC/wgEncodeBroadChipSeq/wgEncodeBroadChipSeqPeaksGm12878H3k36me3.broadPeak.gz>

Since we wanted to use uniform peaks of the transcription factor binding sites from GM12878 and not the clustered track available in the older hg18 release, we downloaded the hg19 data for (2012 freeze). Since multiple centers generated datasets, when multiple datasets were available for a give TF, we only considered data generated using the same antibody. We performed a complete overlap of these datasets and used only the peaks that were common to all datasets. For the TF YY1 however, we took a union of the datasets since there was very little agreement between the two available datasets. The URL (for the data for all TFBs) and the individual datasets (which were merged as described) are as below -

<http://genome.cse.ucsc.edu/cgi-bin/hgFileUi?db=hg19&g=wgEncodeAwgTfbsUniform>

CTCF:

wgEncodeAwgTfbsBroadGm12878CtcfUniPk.narrowPeak

wgEncodeAwgTfbsUtaGm12878CtcfUniPk.narrowPeak

wgEncodeAwgTfbsUwGm12878CtcfUniPk.narrowPeak

EBF1:

wgEncodeAwgTfbsHaibGm12878Ebf1sc137065Pcr1xUniPk.narrowPeak

wgEncodeAwgTfbsSydhGm12878Ebf1sc137065UniPk.narrowPeak

P300:

wgEncodeAwgTfbsHaibGm12878P300Pcr1xUniPk.narrowPeak

wgEncodeAwgTfbsSydhGm12878P300bUniPk.narrowPeak

EP300 - wgEncodeAwgTfbsSydhGm12878P300bUniPk.narrowPeak.gz

PAX5:

wgEncodeAwgTfbsHaibGm12878Pax5c20Pcr1xUniPk.narrowPeak

wgEncodeAwgTfbsHaibGm12878Pax5n19Pcr1xUniPk.narrowPeak

POL2:

wgEncodeAwgTfbsHaibGm12878Pol2Pcr2xUniPk.narrowPeak

wgEncodeAwgTfbsSydhGm12878Pol2IggmusUniPk.narrowPeak

wgEncodeAwgTfbsSydhGm12878Pol2UniPk.narrowPeak

wgEncodeAwgTfbsUtaGm12878Pol2UniPk.narrowPeak

RAD21:

wgEncodeAwgTfbsHaibGm12878Rad21V0416101UniPk.narrowPeak

wgEncodeAwgTfbsSydhGm12878Rad21IggrabUniPk.narrowPeak

YY1:

wgEncodeAwgTfbsHaibGm12878Yy1sc281Pcr1xUniPk.narrowPeak

wgEncodeAwgTfbsSydhGm12878Yy1UniPk.narrowPeak

We then used this compiled dataset to perform a LiftOver (UCSC Genome Browser) into hg18. The overlap between CNV-mQTLs and each of the ENCODE datasets ^1^ was determined by using a 10-base reciprocal overlap. Next, we determined the statistical significance of this overlap by performing 1000 random permutations. Briefly, we determined the size distribution of the CNV-mQTLs by chromosome and generated random datasets from the whole genome matching the CNV-mQTL distribution per chromosome. The distribution of this dataset was used to compute significance of the overlap obtained with the CNV-mQTL dataset.


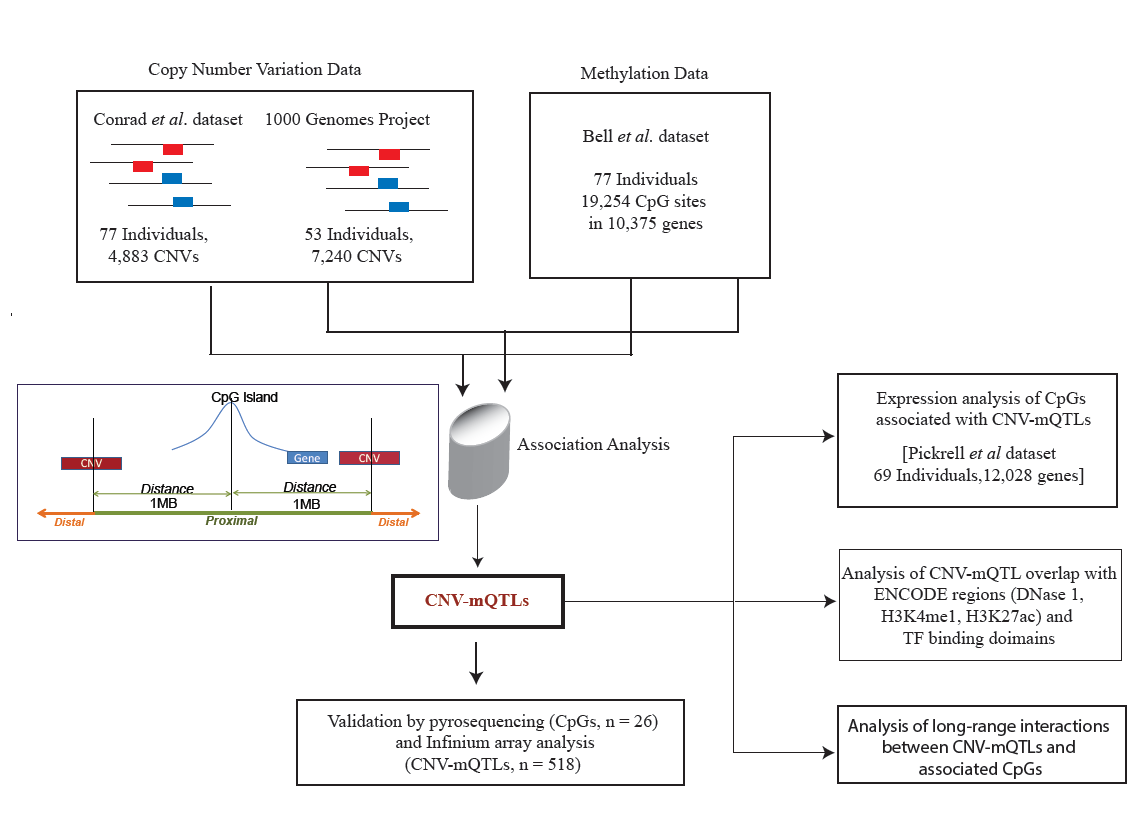


**Fig. S1. Overview of the analysis.** Summary of the analysis pipeline and the datasets used.

**Fig. S2.** Circos plot depicting statistically significant associations between CNV-mQTLs and CpGs (n = 851, permutation test, *P* <0.01). The chromosomes form the outer ring of the Circos plot, followed by genes whose CpG island methylation (blue bars) is associated with CNV-mQTLs (dark orange bars). The association between CNV-mQTLs and CpGs is depicted by arched lines connecting the two, with proximal associations depicted in green and distal associations*,* in orange.

| **a**  **** | **b**  **** |
| --- | --- |

**Fig. S3. Size distribution.** This figure shows that there were no differences in the size distribution of CNV in general and the CNVs that were associated with methylation (CNV-mQTL). The panel on the left (Fig. S3a) shows the size distribution of CNVs, while that on the right (Fig. S3b) shows the size distribution of CNV-mQTLs.

| **a**  **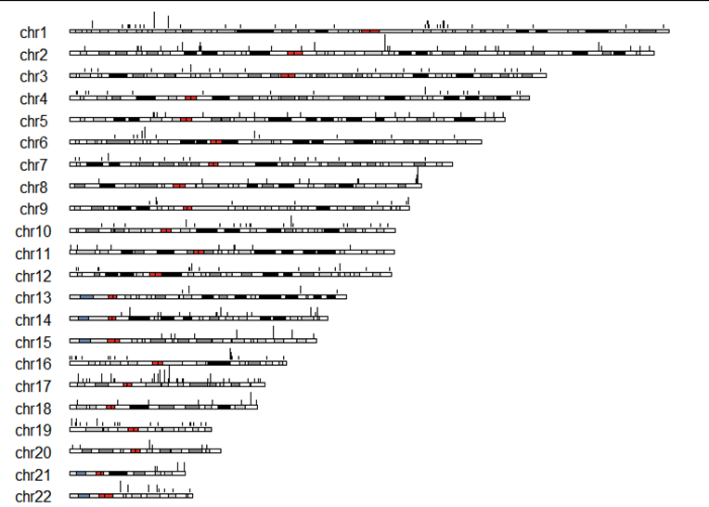** | **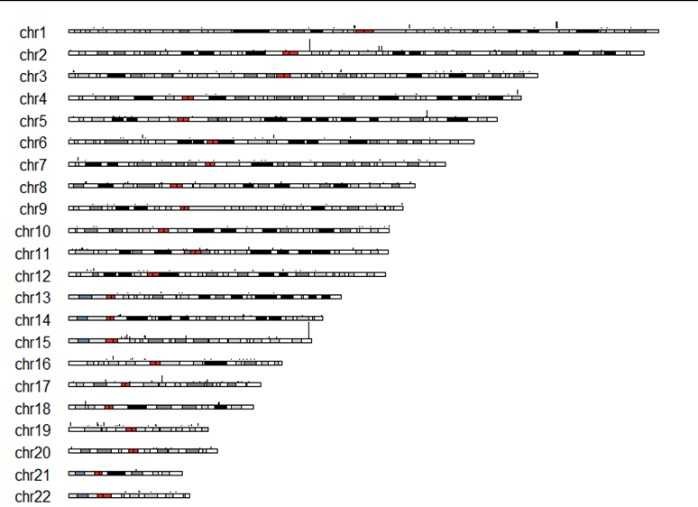** |
| --- | --- |
| **b** | |

**Fig. S4. Overview of CNV-mQTLs. a** Ideograms depicting the distribution of proximal (left) and distal (right) CNV-mQTLs. **b** Gene overlapping CNV-mQTLs were seen to map primarily on introns when compared to exons. This distribution remained the same regardless of mQTLs associated with CpGs that were proximal or distal.

**Fig. S5.** Validation of methylation levels and CNV-mQTL associations. The graphs on left depict the methylation for each sample with varying DNA copy number states (x-axis) as determined by pyrosequencing (blue bars) and Illumina array analysis (red line). The insets on the right show the correlation of copy number (x-axis) and DNA methylation (y-axis) in the discovery cohort. The pyrosequencing data validate the array results of correlation of methylation with DNA copy number.


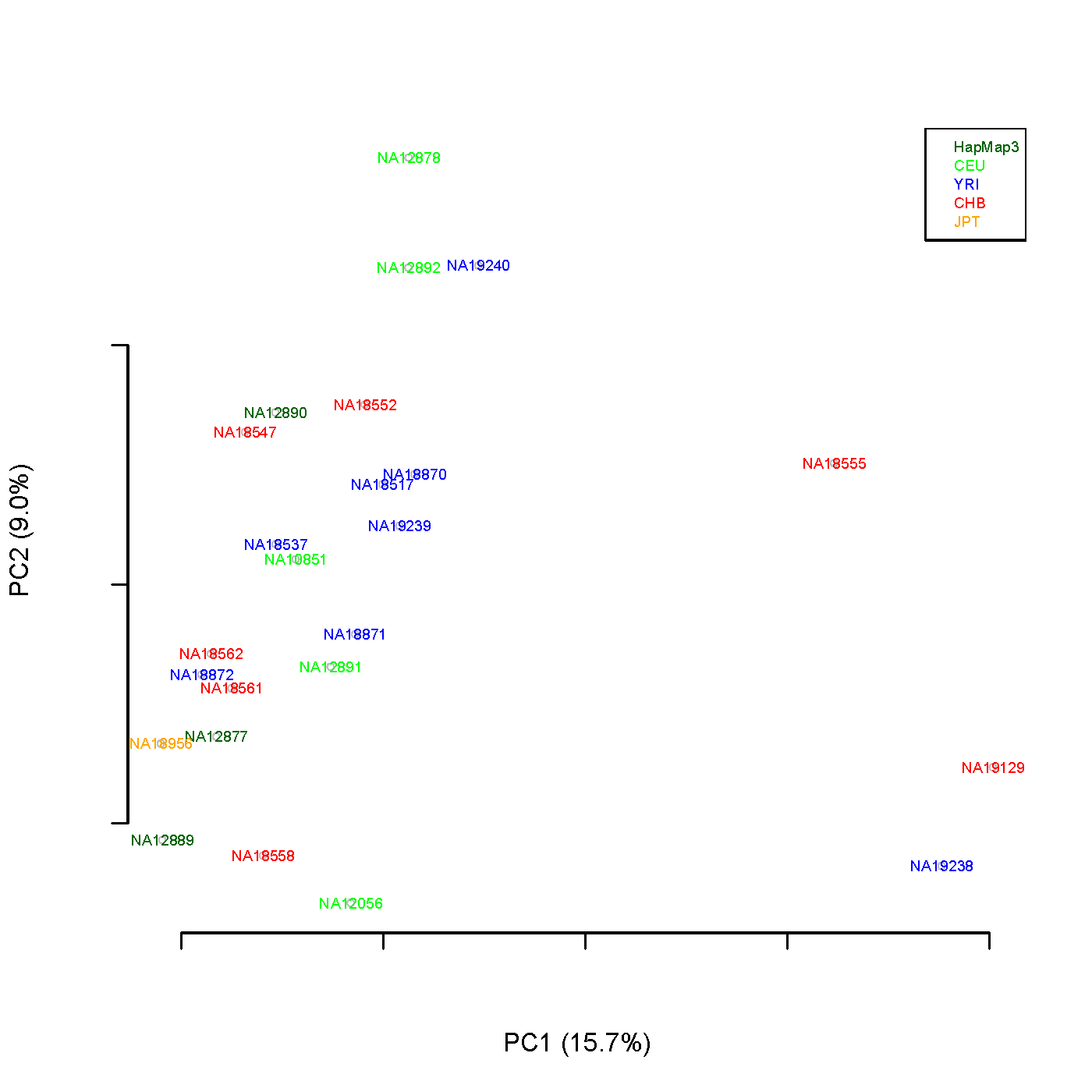


**Fig. S6.** Principal component analysis of the Illumina Methylation 27 array data derived from the 24-sample validation dataset.

**a b**

| **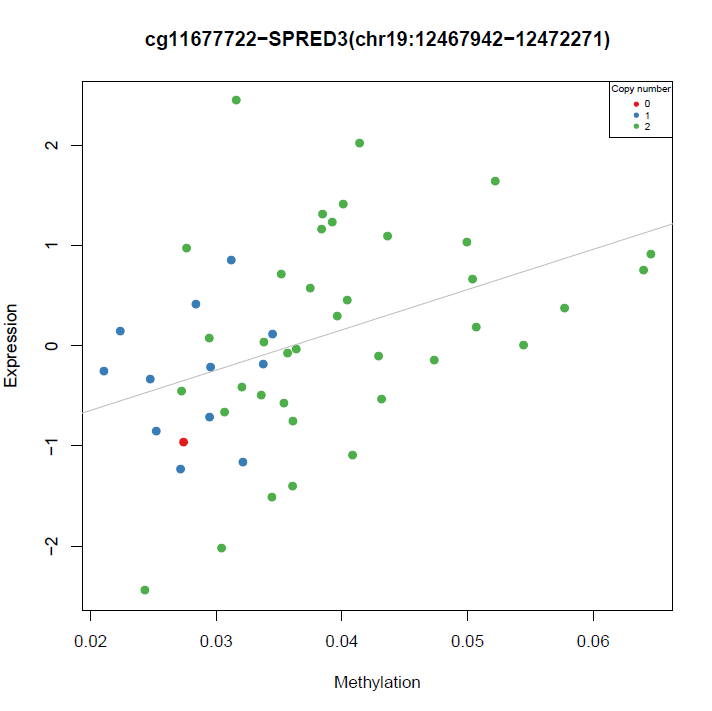** | **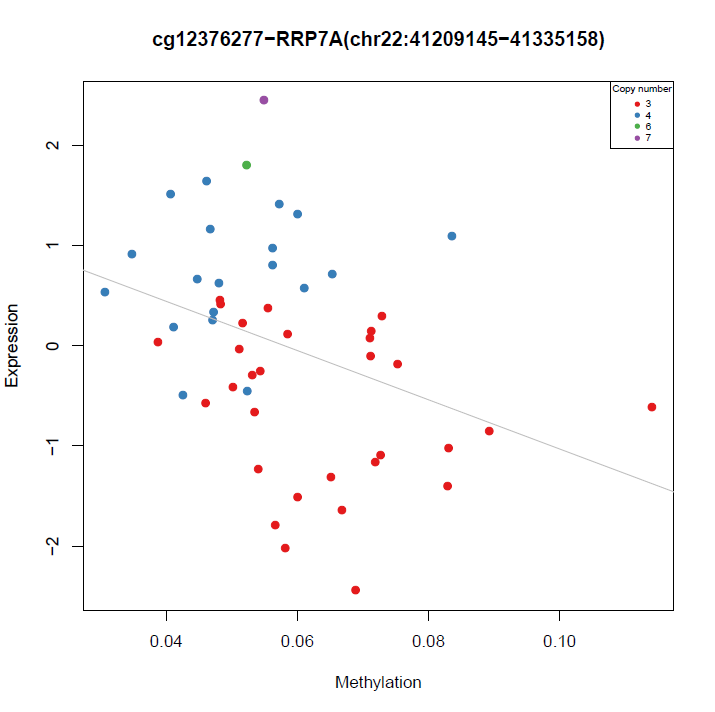** |
| --- | --- |

**Fig. S7. Association with expression. a** Proximal eQTM showing positive correlation between methylation of CpG island cg1677722 of *SPRED3* gene and its expression. The different copy number states of the CNV-mQTL “chr19:12467942-12472271” is depicted. **b** Distal eQTM showing negative correlation between methylation of CpG island, cg12376277 of *RRP7A* and its expression. The different copy number states of the CNV-mQTL “chr22: 41209145-41335158” is depicted.

**Fig. S8.** Percentage of direct overlap of CNV-mQTLs with the various ENCODE regulatory regions. The distributions remained the same when split by proximally and distally associated CNV-mQTLs.

| **__** | **__** | **__** |
| --- | --- | --- |
| **__** | **__** | **__** |
| **__** | **__** | **__** |
| **__** | **__** | **__** |

| **__** | **__** | **__** |
| --- | --- | --- |
| **__** | **__** | **__** |
| **__** | **__** | **__** |
| **__** | **__** | **__** |

| **__** | **__** | **__** |
| --- | --- | --- |
| **__** | **__** | **__** |
| **__** | **__** | **__** |
| **__** | **__** | **__** |

| **__** | **__** | **__** |
| --- | --- | --- |
| **__** | **__** | **__** |
| **__** | **__** | **__** |
| **__** | **__** | **__** |

| **__** | **__** | **__** |
| --- | --- | --- |
| **__** | **__** | **__** |
| **__** | **__** | **__** |

**Fig. S9.** Histograms depicting the normal distribution of randomly permutated data (1000 iterations) and the significance of observed mQTL overlap with various ENCODE datasets as indicated. The red line shows the observed overlaps.

| a | b |
| --- | --- |
| 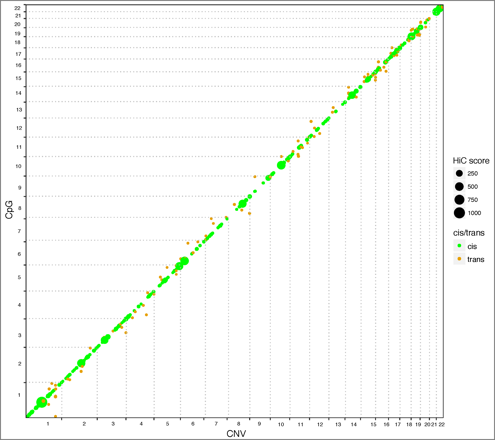 | 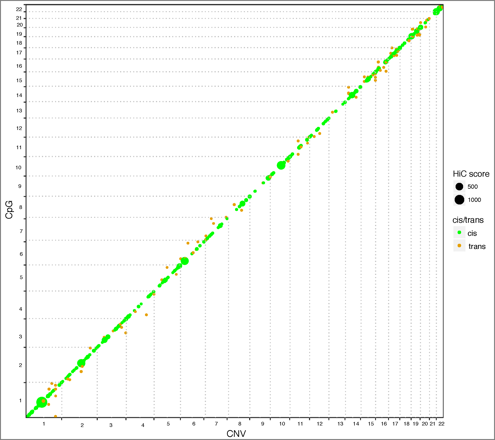 |
|  |  |
| c | d |
| 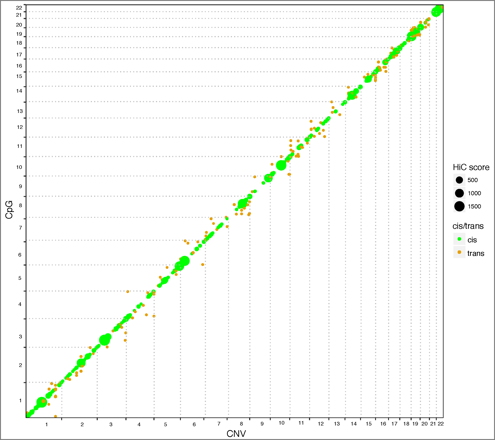 | 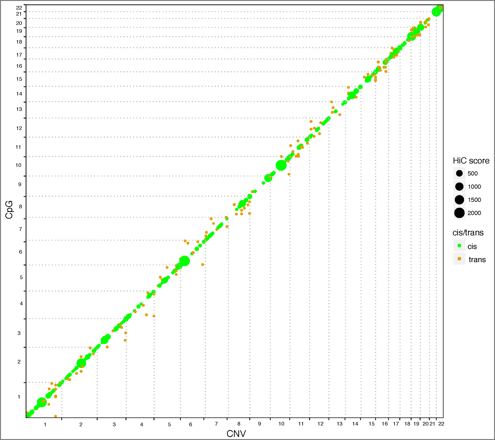 |
|  |  |

**Fig. S10.** Hi-C interaction score plot for CNV-mQTLs that overlap with Hi-C compartments. **a** Overlap of mQTLs and associated CpG regions (with 5 kb window to include shores regions) and Hi-C compartments at 5 kb resolution. **b** Overlap of CNV-mQTLs and associated CpG regions (with 2 kb window to include shores regions) and Hi-C compartments at 5 kb resolution. **c** Overlap of CNV-mQTLs and associated CpG regions (with 5 kb window to include shores regions) and Hi-C compartments at 10 kb resolution. **d** Overlap of CNV-mQTLs and associated CpG regions (with 2 kb window to include shores regions) and Hi-C compartments at 10 kb resolution. The dashed lines denote chromosome separations for 22 autosomal chromosomes.

|  |
| --- |

# **Table S1. Distribution of CNV-mQTL lengths**

| **Size** | **<=1kb** | **1kb~10kb** | **10kb~100kb** | **100kb~1Mb** | **>1Mb** |
| --- | --- | --- | --- | --- | --- |
| **Count** | 242 | 350 | 54 | 10 | 0 |

# **Table S2. Statistics of the MAFs of CNV-mQTLs**

| **MAF** | **<=1%** | **1%~5%** | **5%~10%** | **>10%** |
| --- | --- | --- | --- | --- |
| **Count** | 19 | 70 | 127 | 440 |

# **Table S3. Statistics of the LDs (r^2^) between CNV-mQTLs and nearby SNPs / SNP-mQTLs**

| **LD** | **0~0.1** | **0.1~0.2** | **0.2~0.3** | **0.3~0.4** | **0.4 ~ 0.5** | **0.5~1.0** |
| --- | --- | --- | --- | --- | --- | --- |
| Number of CNV-mQTLs in LD with neighboring SNPs (1Mb window) | 0 | 4 | 32 | 30 | 35 | 555 |
| Number of CNV-mQTLs in LD with neighboring known SNP-mQTLs (1Mb window) | 332 | 153 | 62 | 35 | 12 | 55 |

# **Table S4. The distance between CNV-mQTLs and CpGs**

| **Distance** | **<1 kb** | **1 kb ~ 10 kb** | **10 kb ~ 100 kb** | **100 kb ~ 1 Mb** | **1 Mb ~ 10 Mb** | **10 Mb ~100 Mb** | **>100 Mb** |
| --- | --- | --- | --- | --- | --- | --- | --- |
| Count | 0 | 13 | 64 | 330 | 63 | 332 | 49 |

**Table S5. CNV-mQTLs associated with methylation of imprinted genes.**

| **CNV Coordinates** | **Interaction** | **Gene** | **CpG Island** | **Location** | **Expressed Allele** |
| --- | --- | --- | --- | --- | --- |
| chr6:119118982-119120620 | Distal | IGF2R | cg14556618 | chr6:160,310,121-160,447,573 | Biallelic |
| chr7:93730519-93730716 | Proximal | PPP1R9A | cg16872560 | chr7:94,374,885-94,763,663 | Maternal |
| chr7:148461681-148461744 | Distal | PEG10 | cg06943865 | chr7:94,123,573-94,136,942 | Paternal |
| chr11:3280127-3282487 | Proximal | PHLDA2 | cg04720330 | chr11:2,906,079-2,907,226 | Maternal |
| chr11:42112726-42113054 | Distal | OSBPL5 | cg23617121 | chr11:3,065,607-3,143,116 | Maternal |
| chr11:48557432-48560877 | Distal | SLC22A18 | cg24528523 | chr11:2,877,527-2,903,052 | Maternal |
| chr11:58351105-58352597 | Distal | H19 | cg13145013 | chr11:1,972,982-1,975,641 | Maternal |
| chr13:48431530-48434856 | Proximal | RB1 | cg03085377 | chr13:47,775,884-47,954,027 | Maternal ^(1)^ |
| chr14:99493641-99493712 | Proximal | DLK1 | cg09873258 | chr14:100,263,006-100,271,212 | Paternal |
| chr14:99493641-99493712 | Proximal | MEG3 | cg16567044 | chr14:100,263,006-100,271,212 | Maternal |
| chr19:22899898-22903253 | Distal | PEG3 | cg20897667 | chr19:62,015,615-62,043,887 | Paternal |
| chr20:56593980-56595020 | Proximal | GNAS | cg25983380 | chr20:56,848,190-56,919,645 | Isoform Dependent |

1. Bernstein, B. E. *et al.* An integrated encyclopedia of DNA elements in the human genome. *Nature* **489**, 57–74 (2012).

# **Table S6. Primers**

| **PYROSEQUENCING PRIMERS** | | | | |
| --- | --- | --- | --- | --- |
| **GENES** | **FORWARD PRIMER** | **REVERSE PRIMER** | **SEQUENCING PRIMER** | **Proximal / Distal** |
| H6PD | GTTGTTTGGTATTTGGGTGTT | AACCCTAATTCCATTTACTATCTTTCT | TTTGGTATTTGGGTGTTTA | Proximal |
| KLK5 | GTTGGTTTTAAATTTTTGGTTTTAAGTGA | CCCCACCATAATCATCTTCTTAACTATACT | CTTCTTAACTATACTAATATAACAA | Proximal |
| VPS37B | TTAGTTGAAGGAGATGGTGTAGAAGA | CCCCAAACCCCCCCATTTCTAAC | GAGATGGTGTAGAAGAT | Proximal |
| ANXA7 | TTGAGATTGGGAGGTTGGTGTA | AAAACCTAAAATCACCTAACAACTC | GGTATTTTTTGGGGATAAAG | Proximal |
| MPHOSPH1 | ATTTTAGTGGAGGTTATTGTTAGGA | AAACACTTCATTACAACCTTTTAACT | GGTAAGTAGAGAGGAATGAGTTATA | Proximal |
| APPBP2 | AGTGGATAGGGGTAGATTTTT | AAAAATCCAAACTTTTACCTAAACCTC | GGGGATAAATTATTATAGGA | Proximal |
| DPROKR2 | GGGTGTGGAAGTTATTGGGATTTG | CCCAACTAAACAATACATTTAATCCACTC | TTGTATTTAGTTTTTTGGAGGTT | Proximal |
| TXNRD1 | GGGTTAATTGTTAGAGTTAGAATGATAAAG | AAACACACTTATTCCAAATTTAATTCC | AGAATGATAAAGTAGAAATTTATT | Proximal |
| ACOT2 | GGTTAAGAGTTTATTAGGGTAGAGTTGT | ACAAACCTAAAAAATAACTTTCCAACATAA | AGGGTAGAGTTGTTTGTTAA | Proximal |
| FLJ43276 | GGTGGTGTTGTTGTTTTTTTAAGTTG | CTCTCCCCCCTACCTCAACTCTAAT | GTTGTTGTTTTTTTAAGTTGTG | Proximal |
| RB1 | AGTTTGTGGGGAATGGTT | CCTAACCCCCCACACAACACAAACA | AGGTATTTAGGGTAAGGG | Proximal |
| EZH1 | GTTTTGGAAGTAGTTTGGGAGGTTGTAT | ATTTACTCACTCACCCTCCATCC | GTTTGGGAGGTTGTATT | Proximal |
| DLK1 | GTTTTTATGGTTAGGGGTATAGGG | CACATACAAACCTACCTAAAACAA | GTGTTTTTTTGGGTATTTAAT | Proximal |
| PMS1 | GTTTTTTGGTTGGAATAGGATGAGTA | CTAAACACCCATAATTTATCATACAT | ATTTTAGGTTTTTATTTTGTGGA | Distal |
| GYPC | TAAAGGTAGAGGAAGAGGGTAGAGAGA | CCTCCCTACTAATTTTCTCTTTTAAACCA | CTAATTTTCTCTTTTAAACCATC | Distal |
| SCNN1B | TTTGTAGGGGTGTGGATGTGA | ACCCCACCTCCCCTCAATACATATCACAAA | ACACTCCATCCCACC | Distal |
| CORIN | GGATGATTTTTTTTAAGTTTAAGAGAGATA | ACCCCTAAAAAATAACTTCTTTAAATACA | AGTTTAAGAGAGATAAATTGAAT | Distal |
| DCC | GGTGAGGTTTAGTAAAATAAGTGGA | AAACCTAAAAACTAACTAATCAAAACTACT | GAGGTTTAGTAAAATAAGTGGAT | Distal |
| MTNR1A | TTTATTATAGTGGTGGATATTTTGGGTAA | ACCAAATACTTAAAAAAAACTAACTACC | GGTGGATATTTTGGGTAAT | Distal |
| OSBPL5 | ATTGGTTTTGTTGTTTTTTGTTTTTGTAGG | ACTAAACCTCTCACCTTTACAAT | GTTGTAGGAGTTGTTGGA | Distal |
| RAD9A | AGGAAGGGGTGAGGGGTTA | ACAACTTTCCTCAATTCACACAC | GGGGTGAGGGGTTAG | Distal |
| MSX1 | GGAGGGTGTATTGTGGTTAG | ACACTCCCCCCCCAACAACATCAA | GGGGTTCTTTTATTTTAATGGGT | Distal |
| NEIL3 | AGAGGATTAGATTAGAAAATGTGTGTATA | ATAATTCACCCCCCCCCTAAATCTTACT | GTAAAGAAAATATATTTAGAGTT | Distal |
| CLDN11 | GGTATTGTTTAGTTTAGGTTTAGGTATAGT | AAAACCCAACTCATCCAACTT | CACCACCCAATCATTAATA | Distal |
| FZD3 | GGGGTGTTTAAGAGGTAGATT | AAAAAAACATTTCCTAAAAAACAAAAATTA | TAGAGTAGAGGATAGAATAGGTTAT | Distal |
| ABHD9 | GGTTTTAAGTTTAGTATAGGATTTGTTAGG | ATCCCCCTTAACCTAAAACCCCTCC | GTTTTTGTAGAGTTTAGGTGTT | Distal |
| LEO1 | ATAATGGAGGATATGGAGGATTTT | ATCTAACTCTCCATCCTACCC | ATTATCCCACTCCCAC | Distal |

# **Table S7. Validation Samples**

| **Sample ID** | **Gender** | **Population** | **Relationship** |
| --- | --- | --- | --- |
| NA10851 | Male | CEPH |  |
| NA12056 | Male | CEPH |  |
| NA12877 | Male | CEPH |  |
| NA12878 | Female | CEPH | Child |
| NA12889 | Male | CEPH |  |
| NA12890 | Female | CEPH |  |
| NA12891 | Male | CEPH | Father |
| NA12892 | Female | CEPH | Mother |
| NA18517 | Female | Yoruba |  |
| NA18537 | Female | Yoruba |  |
| NA18547 | Female | Han Chinese |  |
| NA18552 | Female | Han Chinese |  |
| NA18555 | Female | Han Chinese |  |
| NA18558 | Male | Han Chinese |  |
| NA18561 | Male | Han Chinese |  |
| NA18562 | Male | Han Chinese |  |
| NA18870 | Female | Yoruba |  |
| NA18871 | Male | Yoruba |  |
| NA18872 | Male | Yoruba |  |
| NA18956 | Female | Japanese |  |
| NA19129 | Female | Han Chinese |  |
| NA19238 | Female | Yoruba | Mother |
| NA19239 | Male | Yoruba | Father |
| NA19240 | Female | Yoruba | Child |

**References:**

1. Bernstein, B. E. *et al.* An integrated encyclopedia of DNA elements in the human genome. *Nature* **489**, 57–74 (2012).
